# Upcycled Green Coffee Phenolic‐Rich Extract Modulates Key Pathways of Glucose Absorption in Caco‐2 Cells: Findings From a Screening of Upcycled Agro‐Industrial By‐Products for Application in Functional Foods

**DOI:** 10.1002/mnfr.70353

**Published:** 2025-12-08

**Authors:** Nathalia Almeida Costa, Gabriela de Matuoka e Chiocchetti, Maria Carolina Ximenes de Godoy, Alessandra Gambero, Gabriela Alves Macedo, Juliana Alves Macedo

**Affiliations:** ^1^ Food Science and Nutrition Department Faculty of Food Engineering Universidade Estadual de Campinas—UNICAMP Cidade Universitária Zeferino Vaz Campinas Brazil; ^2^ School For Life Sciences, Pontifical Catholic University of Campinas—PUC‐Campinas Campinas Brazil

**Keywords:** Caco‐2, green coffee, intestinal absorption, orange by‐products, peanut skin

## Abstract

While some purified phenolic compounds are known to modulate intestinal carbohydrate metabolism, the potential of complex phenolic‐rich extracts from different agro‐industrial by‐product sources remains underexplored. This study evaluated extracts obtained from major Brazilian by‐products as a sustainable strategy to recover different bioactive compounds with the potential to reduce the glycemic impact of sugars and promote metabolic health. Phenolic‐rich extracts from green coffee (GCE), orange by‐products (OBE), and peanut skin (PSE) were assessed for cytotoxicity potential, sucrase‐isomaltase (SI) inhibition, basolateral glucose transport, gene expression of glucose transporters, and genes involved in glucose transporter 2 (GLUT2) translocation in Caco‐2 cells. The extracts showed no cytotoxicity, except for PSE above 30 µg/mL. OBE and PSE were unable to inhibit SI, and PSE did not inhibit glucose transport and gene expression. GCE inhibited SI activity by up to 51% and achieved maximum inhibition at 100 µg/mL, with no further effect at 10‐fold higher concentrations. Despite not reducing glucose transport, GCE downregulated SGLT‐1 (0.35‐fold) and PKC (0.37‐fold), suggesting a modulatory effect on active glucose transport and possibly interference in GLUT2 translocation. These findings indicate the potential of GCE to modulate intestinal absorption of carbohydrates, making it promising for application in functional foods with possible regulation of the glycemic response.

## Graphical abstract

This study explored the effects of phenolic‐rich extracts from green coffee, orange by‐products, and peanut skin in intestinal carbohydrate metabolism. The extracts were assessed for cytotoxic potential, sucrase‐isomaltase inhibition, glucose transport, and gene expression in a Caco‐2 cell model. The most significant effect was observed with green coffee extract, which was associated with significant sucrase‐isomaltase inhibition and downregulation of sodium‐dependent glucose co‐transporter‐1 (SGLT‐1) and protein kinase C (PKC).

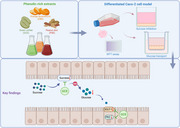

**Abbreviations**
DE: dry extract
FBS: fetal bovine serum
GAE: gallic acid equivalents
GCE: green coffee extract
GLUT2: glucose transporter 2
mRNA: messenger RNA
OBE: orange by‐products extract
PKA: protein kinase A
PKC: protein kinase C
PLC: phospholipase
PSE: peanut skin extract
SGLT1: sodium‐dependent glucose co‐transporter‐1
T2DM: type 2 diabetes mellitus.

## 1 Introduction

The production and processing of food are deeply associated with waste generation. Fruit and vegetable production is responsible for around 45% of food losses worldwide [1]. Sustainable approaches focused on mitigating food losses have used agro‐industrial by‐products as an alternative for recovering bioactive compounds that can be used in food, pharmaceutical, and cosmetic products with potential functionality [2]. Given the abundance of phenolic compounds in non‐consumable plant parts, such as stems, leaves, shells, skins, peels, pomace, and seeds, food industry by‐products can be a significant source for the recovery of these compounds [3].

The bioactivity of phenolic compounds is related to their antioxidant effects, potentially reducing oxidative stress and inflammation, cardioprotective and neuroprotective effects, anti‐cancer, as well as anti‐glycation and anti‐hyperglycemic effects [4, 5, 6]. Considering the benefits associated with it, increased polyphenol intake has emerged as an alternative for regulating intestinal carbohydrate metabolism, since Western diet pattern are characterized by substantial amounts of simple sugars and are linked with non‐communicable diseases such as type 2 diabetes mellitus (T2DM) and obesity [7]. High consumption of sucrose‐based foods is associated with rapid increases in postprandial blood glucose levels, leading to a high glycemic response and increasing the risk of metabolic disorders and progression of clinical complications related to T2DM [8]. Strategies to reduce the glycemic impact of such products are therefore of considerable interest in the development of functional foods.

Phenolic compounds can interfere at various stages of intestinal carbohydrate metabolism, through interaction of polyphenols and carbohydrates causing resistance to digestion; inhibition of digestive enzymes such as α‐glucosidases (sucrase, maltase, and isomaltase) and α‐amylase; decrease in glucose uptake and absorption through inhibition of sodium‐dependent glucose co‐transporter‐1 (SGLT‐1) and glucose transporter glucose transporter 2 (GLUT2); and modulation of gut microbiota [4, 9, 10].

The upcycling of by‐products from coffee, orange, and peanut processing chains to produce phenolic‐rich extracts has shown potential in regulating carbohydrate metabolism. Intake of green coffee extracts (GCEs) [11, 12, 13], orange by‐product extracts (OBEs) [14, 15], and peanut skin extracts (PSEs) [16, 17] has been associated with reduced postprandial glycemia and improved glucose tolerance. The mechanisms underlying the effects of these extracts on the intestinal absorption of sugars have been investigated, especially regarding the inhibition of digestive enzymes by GCEs [18, 19], flavonoids and extracts from orange by‐products [20, 21, 22], as well as procyanidins and extracts from peanut skin [23, 24, 25, 26]. Besides, peanut skin procyanidins are associated with glucose transport inhibition with downregulation of SGLT‐1 and GLUT‐2 [25, 27]. GCE has also been associated with a decrease in glucose uptake and inhibition of glucose transporters expression [28].

Although the effects of purified phenolic compounds on carbohydrate digestion and absorption are well established, the potential of whole phenolic‐rich extracts derived from agro‐industrial by‐products remains poorly explored. In this study, we evaluated extracts obtained from major Brazilian food processing residues for their capacity to modulate the glycemic response to sugars commonly used in food systems. This approach emphasizes the direct valorization of by‐products, such as low‐quality green coffee, orange by‐products, and peanut skin, through simple and scalable extraction methods. In contrast to purified phenolic compounds, these crude extracts can exhibit relevant biological activity without the need for further refinement or solvent‐intensive purification processes.

In our previous study, both GCE and PSE decreased free glucose levels in solutions, either alone or in combination, demonstrating an additive effect. Additionally, PSE strongly inhibited α‐glucosidase, while both PSE and OBE suppressed α‐amylase activity [29]. In this work, we further investigated the effects of these agro‐industrial by‐products extracts on sucrase‐isomaltase (SI) activity, glucose transport, and modulation of genes involved in glucose absorption in a Caco‐2 cell model.

To the best of our knowledge, this is the first report investigating the impact of low‐quality green coffee, orange by‐products, and PSEs on carbohydrate metabolism. The findings support an innovative upcycling strategy for developing sustainable, health‐promoting food ingredients from underutilized agro‐industrial sources.

## 2 Experimental Section

### 2.1 Production of Phenolic‐Rich Extracts From Agro‐Industrial By‐Products

The low‐quality green coffee samples are from *Coffea arabica* species and consist of non‐conforming and defective beans (broken, immature, and black). The orange by‐products (*Citrus sinensis*) are derived from juice extraction, consisting of peels, seeds, and pomace. The peanut skin samples are from the *Arachis hypogaea* species, high oleic Runner variety, from the industrial processing of peanut blanching.

The process for obtaining phenolic‐rich extracts from low‐quality green coffee beans, orange juice industrial by‐products, and peanut skins has been reported previously [29]. Briefly, the phenolic‐rich extracts were obtained by hydroethanolic extraction (50% ethanol, v/v) at a ratio of 1 g of by‐products to 25 mL of solvent. The solutions were subjected to an ultrasonic bath (Ultrasonic Cleaner USC‐1800A, Unique, São Paulo, Brazil) at a frequency of 40 kHz at 30°C for 15 min and subsequent agitation at 200 rpm and 25°C for 15 min in an orbital shaker (MaxQ 8000, Thermo Fisher Scientific, Waltham, USA). The extracts were vacuum‐filtered through 90 mm filter paper and concentrated in a rotary evaporator at 50°C until the organic solvent was completely removed. The concentrated extracts were freeze‐dried for 48 h and frozen at −20°C until subsequent analysis.

### 2.2 Cell Culture

Caco‐2 cells were cultured with high glucose DMEM (Thermo Fisher Scientific, Waltham, USA) supplemented with 10% (v/v) Fetal bovine serum (FBS) (Thermo Fisher Scientific, Waltham, USA), 1% (v/v) Minimal essential medium/Non‐essential amino acids (MEM/NEAA) (Thermo Fisher Scientific, Waltham, USA), called DMEM 10% FBS for now on, and 1% (v/v) penicillin‐streptomycin (Thermo Fisher Scientific, Waltham, USA), in 75 cm^2^ flasks, at 37°C and 5% CO_2_. Culture medium changes were performed on alternating days, and cells were passaged at 70%–80% confluence. Cell passages between 30 and 45 were used in the experiments.

### 2.3 Cell Viability Assay in Caco‐2 Cells

The phenolic‐rich extracts of low‐quality green coffee (GCE), orange by‐products (OBE), and peanut skin (PSE) were evaluated for their cytotoxic potential in Caco‐2 cells using the MTT (3‐[4, 5‐dimethylthiazol‐2‐yl]‐2,5‐diphenyl‐tetrazolium bromide) (Sigma–Aldrich, Darmstadt, Germany) cell viability assay [30]. Caco‐2 cells were seeded (3 × 10^4^ cells/well) in 96‐well plates (Corning Incorporated, Kennebunk, USA) and cultured with DMEM 10% FBS for 24 h (37°C, 5% CO_2_). Subsequently, the cells were exposed to the extracts (1000–5 µg/mL) diluted in DMEM supplemented with 1% FBS, followed by incubation for 24 h. After this period, the sample solution was aspirated, and 100 µL of MTT solution (0.5 mg/mL) diluted in PBS (Thermo Fisher Scientific, Waltham, USA) was added. After 2 h of incubation, the solution was discarded, and 100 µL of DMSO (Êxodo Científica, Sumaré, Brazil) was added to solubilize the formazan crystals completely. Finally, absorbance was measured at 570 nm, and the results were expressed as a percentage of viable cells relative to the control (i.e., cells that had not been exposed to the treatment).

### 2.4 Sucrase‐Isomaltase Inhibition in Caco‐2 Cells

The effect of GCE, OBE, and PSE on SI inhibition was determined using a previously described method [31] with modifications. Caco‐2 cells were seeded at a concentration of 1 × 10^5^ cells/mL in 24‐well plates (Corning Incorporated, Kennebunk, USA) in DMEM 10% FBS, and cultured for 21 days (37°C/5% CO_2_) to form an epithelial monolayer. This cellular assay reflects brush‐border SI activity.

Before the treatment, the cells were washed with 1 mL of PBS, twice. Samples were diluted in PBS and added according to the following groups: Blank (0.5 mL of PBS); negative control (0.5 mL of 10 mg/mL sucrose solution); positive control (0.5 mL of 180 µg/mL acarbose with 10 mg/mL sucrose solution); and samples (0.5 mL of 1000 µg/mL GCE or 1000 µg/mL OBE or PSE 30 µg/mL with 10 mg/mL sucrose solutions). After 2 h of incubation, the supernatant was collected for further glucose quantification using a commercial kit (Bioclin, Belo Horizonte, Brazil). The cells were washed twice with PBS and collected for the determination of protein content [32] to normalize the glucose content. Results were expressed as milligrams of glucose released into the medium/milligrams of total protein.

### 2.5 Glucose Transport in Caco‐2 Cells

The potential of GCE and PSE to inhibit glucose transport was determined by the method of Li et al. [31] with modifications. Caco‐2 cells were seeded at a concentration of 6.43 × 10^4^ cells/cm^2^ in 6‐well inserts (Thincert, Greiner Bio‐One, Kremsmünster, Austria) and cultured for 14 days (37°C/5% CO_2_). Transepithelial electrical resistance (TEER) measurements were performed at room temperature using a Millicell ERS 3.0 Digital Voltohmmeter to ensure cell differentiation. Only cell monolayers with TEER values ≥400 Ω/cm^2^ were used for the experiments.

Transport assays were initiated by removing the culture medium and washing the monolayer twice with PBS. Samples were diluted in DMEM without glucose and 2 mL was added on the apical side according to the following groups: Negative control (4 mg/mL glucose solution); positive control (100 µg/mL phloridzin or phloretin with 4 mg/mL glucose solutions); and samples (1000 µg/mL GCE or 10 µg/mL PSE with 4 mg/mL glucose solutions). Lucifer Yellow (100 µM) (Thermo Fisher Scientific, Waltham, USA) was added in all conditions to monitor monolayer integrity during incubation. On the basal side of the inserts, 2.8 mL of DMEM without glucose was added. Aliquots were recovered at 120 and 240 min of incubation for glucose quantification using the aforementioned commercial kit, and for Lucifer Yellow measurements (Excitation: 428 nm; Emission: 536 nm).

### 2.6 RT‐qPCR

Caco‐2 cells were seeded at a concentration of 3 × 10^5^ cells/well in 6‐well plates and cultured for 14 days (37°C/5% CO2). Samples (1000 µg/mL GCE or 10 µg/mL PSE with 4 mg/mL glucose) were diluted in DMEM, and 3 mL was added to each well, consistent with the groups tested in the transport assays. After 2 h, the cells were washed with PBS twice, and total RNA was purified using a RNeasy Plus Mini Kit (Qiagen, Hilden, Germany), according to the manufacturer's instructions. cDNA was obtained from 100 ng/µL of total RNA, using the High‐Capacity cDNA Reverse Transcription Kit (Thermo Fisher Scientific, Waltham, USA). The reaction was conducted in a MiniAmp Plus Thermal Cycler (Thermo Fisher Scientific, Waltham, USA).

For mRNA expression of SGLT‐1, GLUT2, PKC, PKA, and PLC genes, RT qPCR was executed in a QuantStudio 1 Real‐time PCR system (Applied Biosystems, Waltham, USA). Reactions were executed in a final volume of 12 µL with 6 µL SybrGreen Master Mix (Thermo Fisher Scientific, Waltham, USA), 1.5 µL of each forward and reverse primer at 10 µmol (Thermo Fisher Scientific, Waltham, USA), and 3 µL cDNA (4 ng/µL). Expression of GAPDH mRNA was employed as a control for data normalization. All primers sequences are described in Table 1. PCR efficiency curves were calculated from a dilution curve covering a 50‐fold concentration range. The RT‐qPCR conditions were 95°C for 10 min followed by 40 cycles: 95°C for 15 s, 60°C for 1 min, 95°C for 15 s, 60°C for 1 min, and 95°C for 15 s. The data were analyzed with the Relative Expression Software Tool—REST 2009 (Qiagen, Hilden, Germany).

**TABLE 1:** Sequence and efficiency of the oligonucleotides used in RT‐qPCR.

Protein	Gene	Primer (5′ → 3′)	Efficiency
Sodium‐dependent glucose co‐transporter‐1	*SGLT1*	F: CGAGATCTTGGTGAAAATGTAGAGC R: GCCCTGGTTTTGGTGGTTG	2.21 ± 0.00
Glucose transporter 2	*GLUT2*	F: AGTTAGATGAGGAAGTCAAAGCAA R: TAGGCTGTCGGTAGCTGG	1.98 ± 0.16
Protein kinase C	*PKC*	F: TGAGGAGGATCGAATGAGAAG R: CAGGACAGGATGGCAAGGA	1.98 ± 0.21
Protein kinase A	*PKA*	F: CCATCAAGGCTATATCCAGGTC R: TGCCTTATTGTAGCCCTTGC	1.83 ± 0.07
Phospholipase C	*PLC*	F: GGCGAGGTGTCAGTGAATGG R: CAGGTCTCCTTTGAATCCATCTC	1.93 ± 0.10
Glyceraldehyde‐3‐phosphate dehydrogenase	*GAPDH*	F: CTGTTGCTGTAGCCAAATTCGT R: ACCCACTCCTCCACCTTTGA	1.90 ± 0.20

### 2.7 Statistical Analysis

Results were presented as means ± standard deviations. Statistical significance was assessed through one‐way ANOVA with Dunnett's post hoc tests (*p* < 0.05). Data analysis and visualization were conducted in GraphPad Prism 8.0 software (San Diego, California, USA).

## 3 Results

### 3.1 Cytotoxicity of Phenolic‐Rich Extracts in Caco‐2 Cells

The phytochemical characterization of GCE, OBE, and PSE, including compositional analysis, total phenolic content, antioxidant capacity, and profile of main phenolic compounds, was previously reported in our published study [29] and is presented in the Supporting Information (Tables S1 and S2). The total phenolic content of GCE, OBE, and PSE was 105.96 ± 8.05, 17.43 ± 2.62, and 401.08 ± 21.31 µg GAE/mg DE. As for antioxidant capacity, measured by ORAC method, PSE showed the highest antioxidant activity (3737.9 ± 246.30 µmol TE/mg DE), followed by GCE (2964.9 ± 163.40 µmol TE/mg DE) and OBE (960.50 ± 104.50 µmol TE/mg DE). The phenolic profile determined by HPLC‐DAD showed that the main phenolic compounds of GCE were chlorogenic acid (141.53 ± 2.98 µg/mg DE) and caffeic acid (9.83 ± 0.26 µg/mg DE); for OBE, the main compounds identified were hesperidin (15.39 ± 1.88 µg/mg DE) and narirutin (3.16 ± 0.16 µg/mg DE); and for PSE was characterized by the presence of epicatechin (7.83 ± 0.20 µg/mg DE) and epicatechin gallate (1.02 ± 0.01 µg/mg DE). Herein, we focus on the biological effects of these phenolic‐rich extracts in Caco‐2 cells.

**FIGURE 1:**
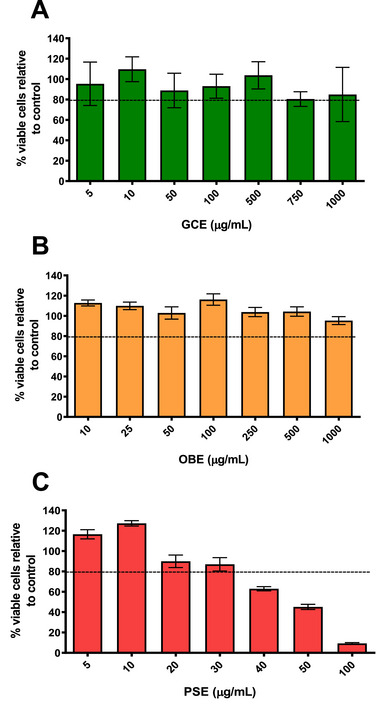
Percentage of cell viability, with respect to control cells, according to the concentration of phenolic‐rich extracts from (A) GCE, (B) OBE, and (C) PSE. Data are expressed as mean ± standard deviation (*n* = 8 biological replicates). The dashed line represents 80% of cell viability. GCE, green coffee extract; OBE, orange by‐products extract; PSE, peanut skin extract.

The assessment of cell viability in Caco‐2 cells after exposure to extracts showed that both GCE and OBE had no cytotoxic effect at concentrations ≤1000 µg/mL, with cell viability above 80% at the concentrations tested (Figure 1). The most significant cytotoxic effect was seen when Caco‐2 cells were exposed to PSE with a safe limit of 30 µg/mL. Therefore, the concentrations selected for subsequent assays were compatible with the maximal non‐cytotoxic doses for this cell line.

### 3.2 Inhibitory Effect of Phenolic‐Rich Extracts From Agro‐Industrial By‐Products on the Sucrase‐Isomaltase Activity in Caco‐2 Cells

**FIGURE 2:**
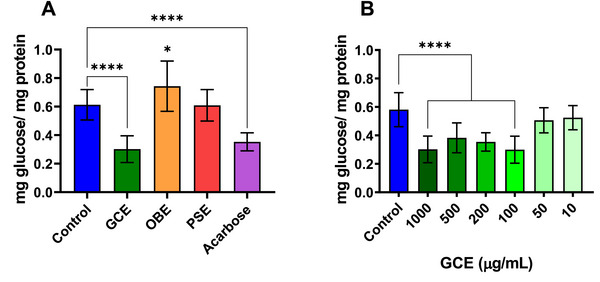
Sucrase‐isomaltase (SI) enzyme activity in differentiated Caco‐2 cells, determined by the glucose released (mg glucose/mg protein) from the enzymatic reaction. (A) Effect of different extracts (GCE and OBE: 1000 µg/mL, PSE: 30 µg/mL) and acarbose (180 µg/mL), compared to the control. (B) Dose‐response effect of GCE on SI inhibition. Values are expressed as mean ± standard deviation (*n* = 8 biological replicates). Asterisks indicate significant differences from control (*p < 0.05; ****p < 0.0001) assessed by ANOVA with Dunnett's post test. GCE, green coffee extract; OBE, orange by‐products extract; PSE, peanut skin extract.

Figure 2A shows the inhibitory effect of GCE, OBE, and PSE on SI activity in differentiated Caco‐2 cells at their respective maximum non‐cytotoxic concentrations. Acarbose (180 µg/mL) was included as a positive control. We observed that the incorporation of GCE at 1000 µg/mL was associated with a significant decrease in glucose derived from sucrose hydrolysis (*p* < 0.0001), corresponding to a 51% inhibition of SI activity (Figure 2A). Used as a positive control, Acarbose also significantly suppressed the enzyme activity of SI, corresponding to a 42% inhibition. In the case of PSE, the tested dose (30 µg/mL) had no inhibitory effect on SI activity compared with the control (*p* = 0.9999). OBE was unable to inhibit SI at a concentration of 1000 µg/mL, whilst elevating glucose levels in the media by 21% (*p* = 0.0189).

GCE remained the only extract to exhibit SI inhibition capacity at the tested concentrations; therefore, its dose‐dependent effect was evaluated (Figure 2B). Caco‐2 cells were exposed to varying concentrations of the extract (10–1000 µg/mL). It was possible to verify that the addition of the extract at concentrations between 100 and 1000 µg/mL significantly suppressed SI activity compared to the control (*p* < 0.0001), with no statistical difference among these concentrations, suggesting an inhibitory effect at a dose 10 times lower than the cytotoxic dose. 10 and 50 µg/mL doses did not show any inhibitory effect.

### 3.3 Effect of Agro‐Industrial By‐Products Extracts on Glucose Transport and Modulation of Glucose Transporters in Caco‐2 Cells

**FIGURE 3:**
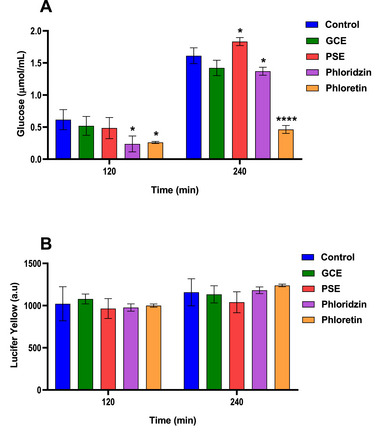
(A) Glucose levels (µmol/mL) in the basal compartment of the inserts after exposure, at the apical side, to different treatments at 120 and 240 min of incubation. (B) Lucifer Yellow levels were detected in the basal compartment of the inserts at 120 and 240 min of incubation. Data represent paracellular permeability and are expressed in arbitrary fluorescence units (a.u). GCE: 1000 µg/mL; PSE: 10 µg/mL; Phloridzin: 100 µg/mL; Phloretin: 100 µg/mL. Values are expressed as mean ± standard deviation (*n* = 3 biological replicates). Asterisks indicate significant differences from control (*p < 0.05; ****p < 0.0001) assessed by ANOVA with Dunnett's post test. GCE, green coffee extract; PSE, peanut skin extract.

The effects of GCE and PSE on glucose transport were investigated in differentiated Caco‐2 cells cultured in inserts to simulate the physiological functions of the human intestinal epithelium. The PSE dose was reduced to 10 µg/mL for transport and relative expression studies because the maximum non‐cytotoxic dose resulted in lower recovery of total RNA after exposure in differentiated Caco‐2 cells (data not shown). The results, expressed as the amount of glucose transported from the apical to the basal compartment of the inserts, are shown in Figure 3A. The levels of lucifer yellow detected in the basal portion confirmed that the monolayers remained intact during the exposure time (Figure 3B).

After 120 min of incubation, treatments with GCE (1000 µg/mL) and PSE (10 µg/mL) resulted in slight decreases in glucose transport of 16% and 21%, respectively. However, these effects were not statistically significant when compared to the control cells. Regarding the positive controls, both phloridzin and phloretin (100 µg/mL) showed a significant effect (*p* = 0.0190 and *p* = 0.0270, respectively) in decreasing glucose levels in the basal compartment by 61% and 58%, respectively, as expected, since phloridzin is a known inhibitor of sodium‐dependent glucose co‐transporter SGLT‐1. In contrast, its aglycone form, phloretin, inhibits the GLUT2 transporter, which mediates the facilitated transport of glucose and fructose [10, 33].

At 240 min of exposure, although a 12% reduction in glucose transport was observed with GCE, this difference was not statistically significant (*p* = 0.0849); conversely, there was a significant 14% increase in glucose transport associated with PSE (*p* = 0.0426). Phloridzin maintained a significant inhibitory effect on glucose transport (*p* = 0.0274) with 15% inhibition. In contrast, phloretin exhibited a strong inhibitory capacity (*p* < 0.0001), resulting in a 71% decrease in glucose levels in the basal compartment.

Table 2 shows the relative expression values of genes involved in glucose transport. The exposure to 1000 µg/mL of GCE reduced SGLT‐1 mRNA to ∼0.35‐fold of control (*p* = 0.024), and reduced PKC mRNA to ∼0.37‐fold of control (*p* = 0.011), suggesting the potential of GCE to modulate the expression of the active glucose transporter, while also acting on PKC gene expression. In the presence of PSE or phloridzin, no significant variations were detected in the relative expression levels of the evaluated genes. On the other hand, phloretin was able to downregulate GLUT2 mRNA to ∼0.45‐fold of control (*p* = 0.032).

**TABLE 2:** Relative expression of genes involved in glucose transport of Caco‐2 cells exposed to different treatments in the presence of glucose (4 mg/mL) after 2 h of incubation, with respect to control cells.

Treatment	SGLT‐1	GLUT2	PKC	PKA	PLC
PSE (10 µg/mL)	−0.628 ± 0.09064	−0.810 ± 0.05607	−0.361 ± 0.03788	−0.356 ± 0.02645	−1.127 ± 0.12545
GCE (1000 µg/mL)	−1.511 ± 0.05742*	−0.610 ± 0.04658	−1.419 ± 0.05092*	−0.830 ± 0.04581	0.130 ± 0.06992
Phloridzin (100 µg/mL)	−0.812 ± 0.10291	−0.969 ± 0.07808	−1.015 ± 0.09581	−0.174 ± 0.04085	−1.363 ± 0.12327
Phloretin (100 µg/mL)	0.006 ± 0.00156	−1.164 ± 0.04022*	−0.025 ± 0.00406	−0.524 ± 0.02841	−1.338 ± 0.10879

*Note*: Values are expressed in log base 2 (mean ± standard deviation, *n* = 3 biological replicates). *Statistically significant down‐regulation compared to the control (*p* < 0.05).

## 4 Discussion

The role of the phenolic‐rich extracts obtained from by‐products of coffee, orange, and peanut production chains in the metabolism of carbohydrates was evaluated in this study according to their ability to inhibit SI activity and glucose transport, as well as their capacity to modulate genes involved in this process in Caco‐2 cells.

The OBE extract had no cytotoxic effect under all concentrations tested (10–1000 µg/mL). A similar result was reported by Barbosa et al. [34] when assessing various doses (25–500 µg/mL) of hydroalcoholic extracts of citrus by‐products. When evaluating SI inhibition in Caco‐2 cells at non‐cytotoxic doses, OBE increased the glucose released from sucrose hydrolysis. This effect, unlike the one we expected, was perhaps due to the hydrolysis of disaccharides present in the extract itself, as OBE had a high carbohydrate content (79%), as reported in our previous study [29].

Unlike our results, other studies reported an inhibition effect on enzymes related to carbohydrate metabolism, as α‐glucosidases and α‐amylase, in the presence of extracts from orange by‐products [20, 21, 22, 29]; however, they only achieved these results at high concentrations (8–25 mg/mL), which exceed the safe dose for Caco‐2 culture, or when compounds isolated from extracts were used. Although orange by‐products are rich in phenolics that can impact carbohydrate metabolism, such as hesperidin and narirutin [22, 35, 36], the dose of these compounds would probably need to be higher to exert an inhibitory and non‐toxic effect on Caco‐2 cells. Perhaps purified extracts could serve as an alternative for future studies on this type of residue. However, in this study, due to the results obtained on SI inhibition, its potential in glucose transport and gene regulation was not evaluated.

Regarding the PSE, the cell viability assay showed that this extract was only viable at low concentrations (up to 30 µg/mL). When assessing the cytotoxic potential of a hydroethanolic PSE in Caco‐2 cells, De Matos et al. [37] found that non‐cytotoxic doses corresponded to lower extract concentrations (≤10 µg/mL). This result was attributed to the high phenolic content of the extract (538 mg GAE/g), coupled with the high pro‐oxidant activity of polyphenols, which could be altering the mitochondrial function in the analyzed cells. This finding is consistent with the data found in the present work. The slight difference in non‐cytotoxic doses between the studies may be attributed to the lower total phenolic content of PSE, corresponding to 401 mg GAE/g dry extract [29]. Future studies are needed to investigate these cytotoxic effects and explore additional beneficial properties of PSE.

Concerning carbohydrate metabolism, PSE did not affect SI inhibition at the tested concentrations in Caco‐2 cells. The phenolic profile of PSE indicated mainly the presence of catechins and procyanidins [29]. Other studies reported the effects of these compounds on enzyme inhibition and glucose transport. Still, this effect was probably not observed in this work due to the lower concentration of the evaluated dose. Kan et al. [38] reported that green tea extract and epigallocatechin gallate inhibited SI by less than 50% at the highest concentrations tested, 2.5 mg/mL and 1 mM, respectively. Peanut skin procyanidins showed no significant effect on SI activity at the tested concentrations (1–10 µg/mL), but the higher dose was associated with increased maltase inhibition and a time‐dependent effect on glucose transport [25]. Procyanidins purified from the aqueous extract of peanut skin showed a dose‐dependent inhibition of rat intestinal SI at concentrations of 1 to 5 mg/mL and glucose transport inhibition in Caco‐2 cells was only achieved at 60 µg/mL [26]. These results show that enzyme inhibition was associated with high concentrations of extracts, whereas the purification of the compounds of interest confers high bioactivity even at low concentrations.

Nevertheless, PSE resulted in a slight yet significant increase in glucose transport, without affecting the relative expression of genes involved in glucose transport. This unexpected behavior may be associated with specific phenolic compounds that affect intestinal transporter function. For instance, Lieder et al. [39] reported that a flavanone compound increased glucose uptake by activating the SGLT‐1 transporter, without evaluating basolateral transport. To the best of our knowledge, there are no reports on the effect of phenolic compounds on increasing basolateral glucose transport in Caco‐2 cells. It is also important to note that our extracts are complex mixtures derived from industrial by‐products and may contain both inhibitory and stimulatory components that act simultaneously. Further studies would be necessary to clarify the underlying mechanism.

In contrast to the OBE and PSE, GCE presented strong SI inhibition, with significant capacity starting at 100 µg/mL. The plateau in GCE inhibitory effect (doses 100 to 1000 µg/mL) may indicate saturation of molecular targets at lower concentrations. This occurs through interaction between phenolic compounds and free enzyme or enzyme‐substrate complex, which may cause conformational changes in SI affecting its catalytic capacity [40]. A concentration‐independent efficacy could be advantageous for therapeutic applications where lower doses may minimize potential toxicity.

The main phenolic compound identified in GCE was chlorogenic acid (141.53 µg/mg dry extract); its hypoglycemic effect has been demonstrated by different mechanisms. Such as inhibition of α‐amylase [41], increased insulin sensitivity, and inhibition of SGLT‐1 and GLUT2 transporters [42, 43]. Our findings align with a previous report where chlorogenic acid‐rich mulberry leaf extract also demonstrated strong SI inhibition in Caco‐2 cells [31]. Chlorogenic acid is formed by ester bonds between *trans*‐cinnamic acids, such as quinic acid and caffeic acid, with 5‐*O*‐caffeoylquinic acid (5‐CQA) being the major isomer found in plants [44]. This compound exhibits high binding affinity (K_i1_) to SI, functioning as a non‐competitive inhibitor [40], likely decreasing the digestion rate of sucrose‐rich foods.

**FIGURE 4:**
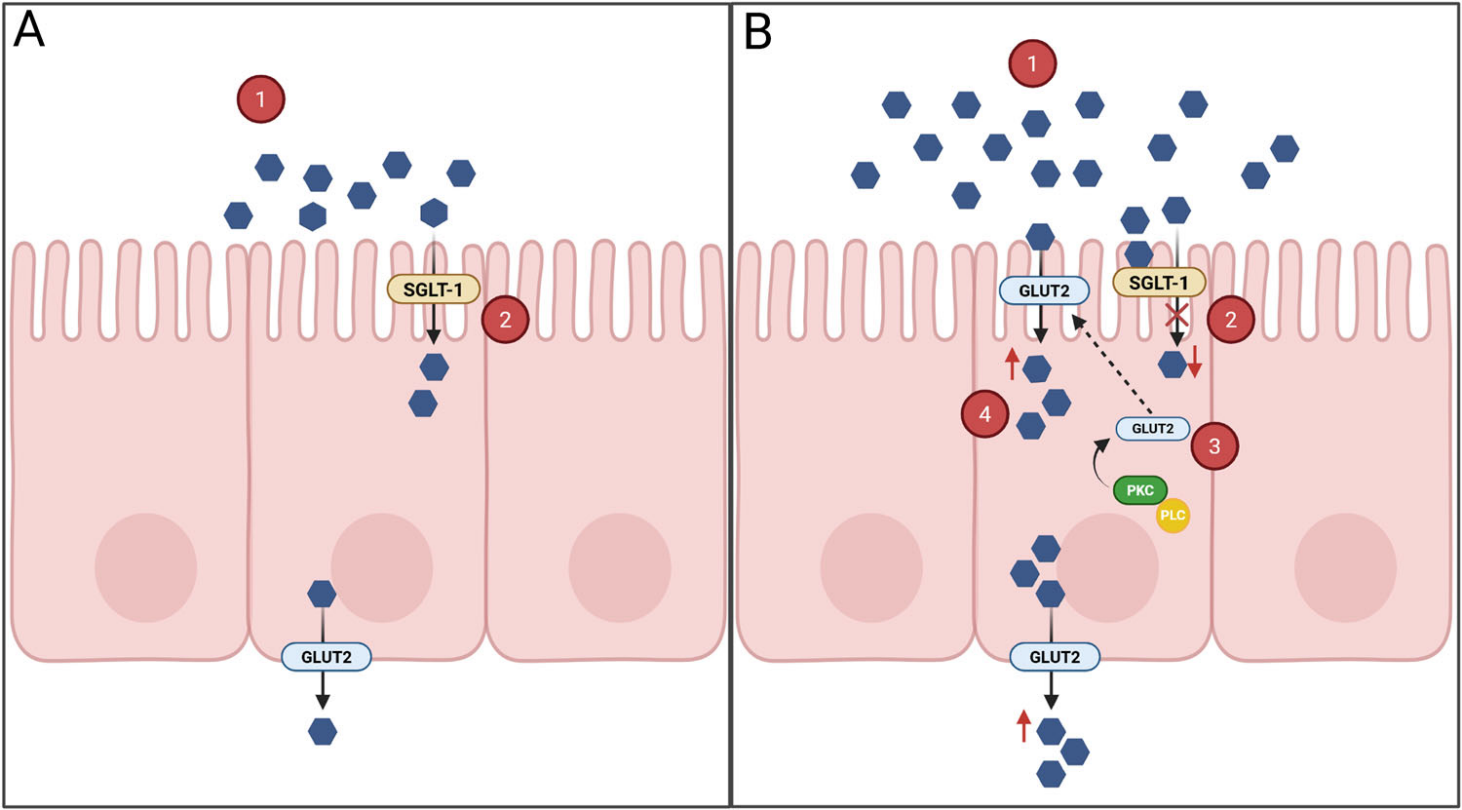
Conceptual diagram of the molecular dynamics of glucose absorption in conditions of (A) lower luminal glucose concentration (<30 mM) and (B) high luminal glucose concentration (≥30 mM). The blue hexagon represents glucose. Panel (A): 1. Lower glucose concentration in the intestinal lumen (≤30 mM); 2. Glucose is mainly absorbed through sodium‐dependent glucose co‐transporter‐1 (SGLT‐1). Panel (B): 1. High glucose concentration in the intestinal lumen (≥30 mM); 2. Saturation of SGLT‐1; 3. Increased translocation of glucose transporter 2 (GLUT2) to the apical membrane through stimulation of protein kinase C (PKC), in the presence of phospholipase C (PLC); 4. Increased glucose absorption through GLUT2. Created in BioRender. DECAN, L. (2025) https://BioRender.com/gidsk1z.

Although GCE did not significantly decrease glucose transport at the tested dose, there was a significant decrease in SGLT‐1 and PKC mRNA expression. Glucose absorption occurs mainly through the active transporter SGLT‐1 (Figure 4), present in the brush border membrane of enterocytes, which has a high affinity associated with a low transport capacity. At high glucose concentrations in the intestinal lumen (≥30 mM), the GLUT2 transporter is translocated to the apical surface to promote facilitated glucose transport, since it is a low‐affinity protein with high transport activity. The GLUT2 translocation process is regulated by the phospholipase C (PLC) and protein kinase C (PKC) signaling pathways [33, 45, 46]. The results indicate that GCE decreased SGLT‐1 gene expression without significantly affecting transporter activity at the apical membrane. Besides, by decreasing PKC gene expression, GCE may play a role in slowing the migration of GLUT2 to the apical surface. The absence of changes in GLUT2 expression after 2 h of treatment may be related to the short exposure time, since this transporter often shows delayed transcriptional responses. Peixoto et al. [28] also verified a decrease in SGLT‐1 mRNA expression levels in the presence of green coffee aqueous extract, at the same concentration used in the present work; however, by evaluating prolonged exposure over 24 h, they also detected a decrease in GLUT2 mRNA expression.

Several studies indicated the potential of chlorogenic acid, isolated or in plant extracts, to inhibit glucose transport in Caco‐2 cells, acting directly on the SGLT‐1 and GLUT2 transporters. Some of these works assessed glucose uptake, which corresponds only to the amount of glucose that crosses the apical membrane entering the enterocytes [28, 47, 48], and others evaluated glucose transport basolaterally [49, 50]. Nonetheless, the use of radiolabeled glucose tracers, which are generally associated with low glucose concentrations and short exposure times, does not reflect the physiological conditions typical of the postprandial state, with high luminal glucose concentration and consequent glucose transport overload [10].

In addition, studies suggest that diabetes is related to greater expression of glucose and fructose transporters, such as SGLT‐1, GLUT2, and GLUT5, thus contributing to an exacerbation of the hyperglycemic response [51, 52, 53, 54]. Phloridzin is a known inhibitor of SGLT‐1, while its aglycone form, phloretin, is able to inhibit the GLUT2 transporter [10]. In this study, the phloridzin treatment was able to slightly decrease glucose transport, but the greatest transport inhibition effect was observed after the addition of phloretin, suggesting that in this model, GLUT2 is primarily responsible for glucose uptake. This may have influenced the response of GCE in glucose transport, given its capacity to inhibit SGLT‐1 expression without significantly altering GLUT2 expression levels.

**FIGURE 5:**
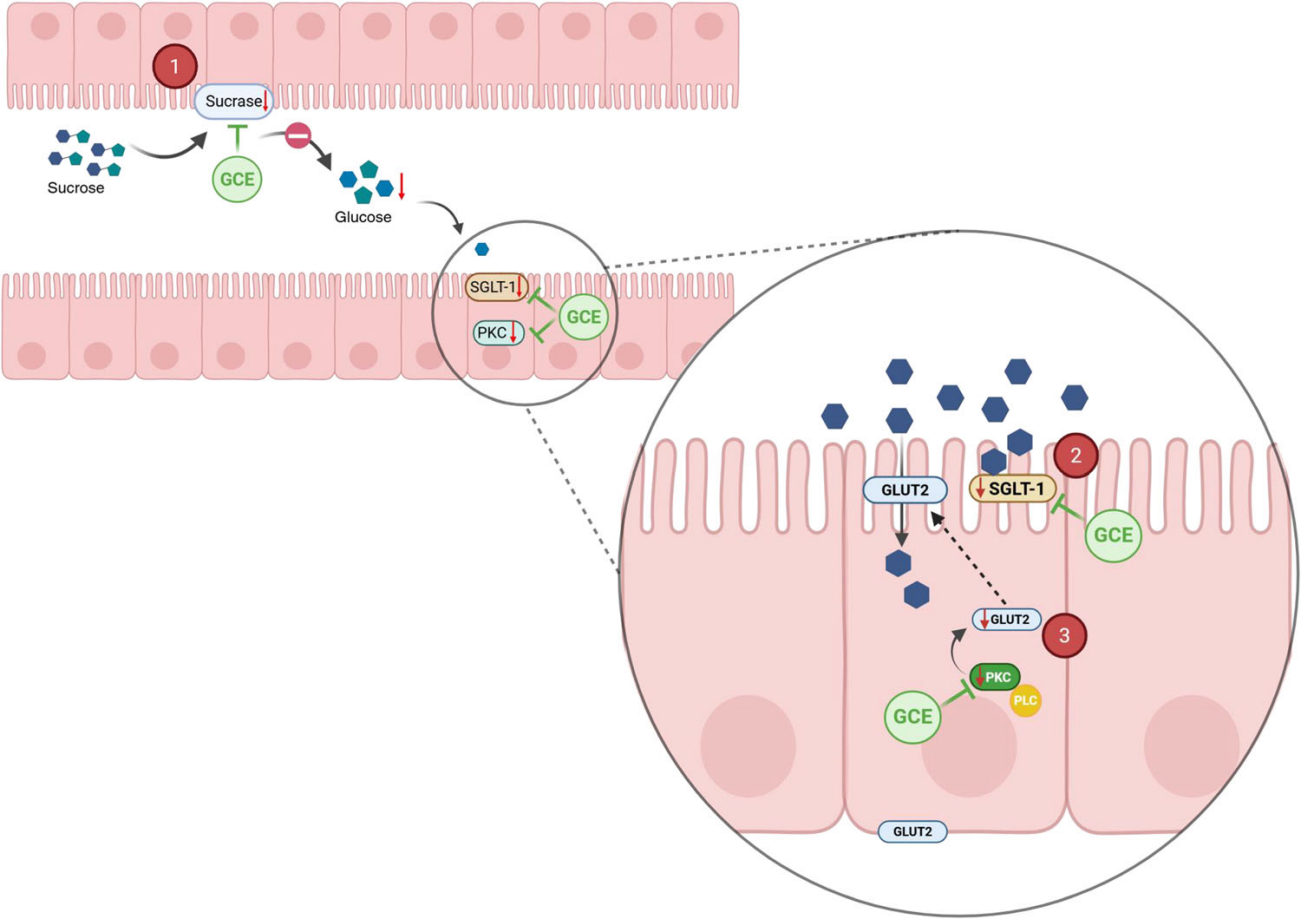
Proposed scheme of the mechanism of action of green coffee extract (GCE) at different stages of sucrose digestion. 1. Inhibition of sucrase‐isomaltase by GCE, resulting in lower glucose release in the intestinal lumen; 2. Downregulation of SGLT‐1 gene expression mediated by GCE; 3. Downregulation of PKC gene expression mediated by GCE and the possible effect on decreasing GLUT2 translocation to the apical membrane. The blue hexagon represents a glucose molecule, and the green pentagon represents fructose. GLUT2, glucose transporter 2; PKC, protein kinase C; PLC, phospholipase C; SGLT‐1, sodium‐dependent glucose co‐transporter‐1. Created in BioRender. DECAN, L. (2025) https://BioRender.com/gidsk1z.

Overall, GCE demonstrated potential to modulate intestinal glucose absorption through two distinct mechanisms (Figure 5). The first is inhibition of SI with a saturation effect, which may directly limit sucrose digestion. The second involves downregulation of SGLT‐1 and PKC gene expression. Given the absence of an inhibitory effect on glucose transport at 120 and 240 min, it is likely that the reduction in SGLT‐1 and PKC mRNA was not characterized by a decrease in protein translation during the evaluated period, suggesting a possible long‐term effect of GCE on the decrease in SGLT‐1 and PKC activity. Based on the observed effects, it is likely that the co‐ingestion of GCE with sucrose would result in a decrease in the breakdown of this disaccharide and, therefore, a lower release of glucose, impacting the glucose content available for absorption. In addition, we hypothesized that prolonged consumption of GCE may be related with a decrease in SGLT‐1 and PKC translation, with a possible impact on the decrease in GLUT‐2 translocation.

However, it should be emphasized that the effects observed with GCE application can also be attributed to other components of the extract, such as dietary fibers. Given that the extracts were evaluated in their integral form, without prior purification, it may be challenging to identify specific active compounds. It is also possible that synergistic interactions among the various phenolic compounds in the extracts contributed to the overall activity, as is commonly reported for complex plant matrices. Therefore, future studies involving the fractionation or purification of the extracts would be valuable for identifying individual compounds and clarifying whether the effects are truly synergistic or primarily driven by specific constituents. Additionally, using a cell culture model limits the range of extract concentrations tested due to possible cytotoxic effects. Despite this, the physiological relevance of the findings increases, as they reflect more accurate exposure conditions.

A summary of the main effects observed for GCE, OBE, and PSE is presented in Table 3. Considering the effects observed after incorporation of the different extracts, it can be concluded that OBE did not exhibit enzyme‐inhibitory activity, indicating a low hypoglycemic potential. PSE was also unable to inhibit SI or reduce glucose transport in Caco‐2 cells, likely due to the low concentrations tested, suggesting the need for further research into its cytotoxic potential. The high capacity to inhibit SI activity, along with strong downregulation of SGLT‐1 and PKC mRNA expression, demonstrates the high hypoglycemic potential of GCE, indicating its potential for application in foods with high sugar content for possible regulation of the glycemic response. In vivo studies and clinical trials are needed to confirm the hypoglycemic effect of GCE.

**TABLE 3:** Overview of the effects on intestinal carbohydrate metabolism observed after exposure to phenolic‐rich extracts from agro‐industrial by‐products on Caco‐2 cells.

	Cell cytotoxicity	SI inhibition	Glucose transport		Glucose transporters modulation	Biological implications
			120 min	240 min		
GCE	No cytotoxic effect at doses ≤1000 µg/mL	51% inhibition at a dose of 1000 µg/mL (*p* < 0.0001)	Non‐significant decrease (16%) at a dose of 1000 µg/mL (*p* = 0.7852)	Non‐significant decrease (12%) at a dose of 1000 µg/mL (*p* = 0.0849)	SGLT‐1 mRNA decreased to 0.35‐fold of control PKC mRNA decreased to 0.37‐fold of control	Decreased sucrose hydrolysis and reduced release of glucose for absorption, potentially causing a long‐term decrease in glucose absorption.
OBE	No cytotoxic effect at doses ≤1000 µg/mL	21% increase at a dose of 1000 µg/mL (*p* = 0.0189)	n.e.	n.e.	n.e.	No hypoglycemic effect at the tested conditions
PSE	No cytotoxic effect at doses ≤30 µg/mL	No inhibition at a dose of 30 µg/mL (*p* = 0.9999)	Non‐significant decrease (21%) at a dose of 10 µg/mL (*p* = 0.5899)	Significant increase (14%) at a dose of 10 µg/mL (*p* = 0.0426)	No significant effects	No hypoglycemic effect at the tested conditions

*Note*: Statistical significancy assessed by ANOVA with Dunnett's post test.

Abbreviations: GCE, green coffee extract; GLUT2, glucose transporter 2; n.e., not evaluated; OBE, orange by‐products extract; PKC, protein kinase C; PLC, phospholipase C; PSE, peanut skin extract; SGLT‐1, sodium‐dependent glucose co‐transporter‐1; SI, sucrase‐isomaltase.

## Conflicts of Interest

The authors declare no conflicts of interest.

## Supporting information




**Supporting File**: mnfr70353‐sup‐0001‐SuppMat.docx.

## Data Availability

The data that support the findings of this study are available from the corresponding author upon reasonable request.
