# Supplementary material for: Upcycled Green Coffee Phenolic‐Rich Extract Modulates Key Pathways of Glucose Absorption in Caco‐2 Cells: Findings From a Screening of Upcycled Agro‐Industrial By‐Products for Application in Functional Foods
Source: Mol Nutr Food Res. 2025 Dec 8;70(1):e70353. doi: 10.1002/mnfr.70353 (PMC12728385; doi:10.1002/mnfr.70353)
Supplement: Supplementary file 1 — Supporting File: mnfr70353‐sup‐0001‐SuppMat.docx. [file MNFR-70-e70353-s001.docx]

**Upcycled Green Coffee Phenolic-rich Extract Modulates Key Pathways of Glucose Absorption in Caco-2 Cells: Findings from a Screening of Upcycled Agroindustrial By-products for Application in Functional Foods**

Nathalia Almeida Costa1*, Gabriela de Matuoka e Chiocchetti1, Maria Carolina Ximenes de Godoy2, Alessandra Gambero2, Gabriela Alves Macedo1, Juliana Alves Macedo1

1 Food Science and Nutrition Department, Faculty of Food Engineering, Universidade Estadual de Campinas – UNICAMP, Rua Monteiro Lobato, 80, Cidade Universitária Zeferino Vaz, CP 6121, CEP 13083-862, Campinas, Brazil.

2 School for Life Sciences, Pontifical Catholic University of Campinas - PUC-Campinas, Av. John Boyd Dunlop, s/n, Jardim Ipaussurama, Campinas, SP, Brazil

* Corresponding author.

E-mail address: nathaliaalmeida.nutri@gmail.com (N. Costa).

The data presented here was previously published:

Nathalia Almeida Costa, Gabriela de Matuoka e Chiocchetti, Bárbara Morandi Lepaus, Julia Millena dos Santos Silva, Flávio Martins Montenegro, Gisele Anne Camargo, Gabriela Alves Macedo, and Juliana Alves Macedo. ACS Food Science & Technology 2025 5 (1), 327-335. DOI: 10.1021/acsfoodscitech.4c00811.

<<https://pubs.acs.org/doi/10.1021/acsfoodscitech.4c00811>>

Notice to readers: Further permissions related to the material excerpted should be directed to the ACS Publications.

**Table S1.** Centesimal composition of agroindustrial by-products and chemical and antioxidant characterization of their respective phenolic-rich extracts.

|  | Green coffee | Orange by-products | Peanut skin |
| --- | --- | --- | --- |
| Centesimal composition of agroindustrial by-products (g/100g) |  |  |  |
| Moisture and volatiles | 4.87 ± 0.06 | 5.91 ± 0.01 | 10.62 ± 0.09 |
| Ash | 4.16 ± 0.03 | 5.89 ± 0.05 | 2.19 ± 0.01 |
| Total fats | 16.33 ± 0.13 | 2.47 ± 0.06 | 20.47 ± 0.06 |
| Protein | 13.82 ± 0.11 | 6.69 ± 0.02 | 15.25 ± 0.05 |
| Soluble fibers | 5.10 ± 0.00 | 19.67 ± 0.24 | ND < 0.10 |
| Insoluble fibers | 42.47 ± 0.27 | 25.47 ± 0.38 | 52.12 ± 0.11 |
| Carbohydrates | 60.82 | 79.04 | 51.47 |
| Energy value (kcal/100g) | 446 | 365 | 451 |
| Extracts characterization |  |  |  |
| Yield (%) | 14.07% ± 1.89 | 25.91% ± 0.96 | 15.37% ± 0.20 |
| Total phenolic content  (µg GAE/mg DE) | 105.96 ± 8.05 | 17.43 ± 2.62 | 401.08 ± 21.31 |
| ORAC  (µmol TE/mg DE) | 2964.9 ± 163.40 | 960.5 ± 104.50 | 3737.9 ± 246.30 |

Values expressed as mean ± standard deviation (n = 3). DE: Dry extract; GAE: Gallic acid equivalent; TE: Trolox equivalent. Notice to readers: Further permissions related to the material excerpted should be directed to the ACS Publications.

**Table S2.** Phenolic profile of the phenolic-rich extracts.

| Extracts | Compounds (µg/mg DE) | |
| --- | --- | --- |
| GCE | Chlorogenic acid | 141.527 ± 2.984 |
|  | Caffeine* | 59.863 ± 2.206 |
|  | Caffeic acid | 9.826 ± 0.258 |
| OBE | Hesperidin | 15.387 ± 1.876 |
|  | Natirutin | 3.159 ± 0.154 |
|  | Tangeretin | 0.200 ± 0.033 |
|  | Hesperetin | 0.135 ± 0.031 |
| PSE | Epicatechin | 7.831 ± 0.199 |
|  | Epicatechin gallate | 1.024 ± 0.010 |
|  | Catechin | 0.538 ± 0.014 |
|  | Procyanidin B1 | 0.431 ± 0.011 |
|  | Procyanidin B2 | 0.353 ± 0.012 |
|  | Epigallocatechin gallate | 0.204 ± 0.009 |
|  | Gallic acid | 0.170 ± 0.007 |
|  | p-Coumaric acid | 0.074 ± 0.000 |

Values expressed as mean ± standard deviation (n = 3). *Not a phenolic compound. GCE: Green coffee extract; OBE: Orange by-product extract; PSE: Peanut skin extract; DE: Dry extract. Notice to readers: Further permissions related to the material excerpted should be directed to the ACS Publications.
